# A Dissertation on the Protective Powers of Vaccinia; Being the Essay to Which Was Unanimously Awarded the Prize of the Boylston Medical Committee of Harvard University for 1844

**Published:** 1844-11

**Authors:** 


### BIBLIOGRAPHICAL NOTICES.

*A Dissertation on the protective powers of Vaccinia; being the Essay to which was unanimously awarded the Prize of the Boylston Medical Committee of Harvard University for* 1844. By Samuel Forry, M. D., Editor of "The New York Journal of Medicine."

This Essay of Dr. Forry is published in the New York Journal of Medicine for Sept. last. It covers 28 pages of that valuable periodical, and is well worthy of the perusal of every member of the profession. It embodies a mass of information upon the subject of the protective powers of Vaccinia, which can scarcely be met with elsewhere. We cannot do better than present to our readers some of the conclusions which are deduced from the evidence thus collated. The questions proposed for the prize essay, above referred to, were as follows:—

"*To what extent is the human system protected from small-pox, by inoculation with the cow-pox?*

"*Is the protection increased by revaccination, and if so, under what circumstances?*"

In answer to the first inquiry, the Dr. remarks in general terms, "*that when perfect, it is as complete a protection as any other prophylactic known to man.* It is a general law that an individual is insusceptible of a second attack of small-pox, and yet cases of recurrent variola are so common, at the present day, as not even to excite our surprise. It is not, however, the less a general law, which, like all other vital laws, is subject to exception. On the other hand, variola after vaccination does also occur; but as this does not happen, when vaccination is properly performed, more frequently than the occurrence of small-pox after small-pox, it follows that the general law is equally active in both cases. Upon this point, however, hangs the whole question; for, if it can be proved, as it doubtless will be in the sequel of this essay, that the occurrence of small-pox after vaccination is not more apt to take place than after variolation, the conclusion that vaccinia secures as complete a protection against variola as any other prophylactic agent known to man, is a legitimate deduction. Perfect vaccination, may, therefore, be considered as equivalent to an attack of small-pox; and vaccinia be regarded as identical with variola, save the greater mildness resulting from its transmission through the cow."

A discussion upon the laws of epidemics, and their applicability to epidemic small-pox follows, with remarks upon *contagion* and *infection*, which we have not room to notice.

The identity of small-pox and vaccinia is considered by the writer to be "demonstrated by experiments scientifically conducted, by infecting the cow with variola, and thus producing the vaccine infection."

"The extent of protection was estimated too highly by the early friends of vaccination; for during its prevalence as an epidemic, its prophylactic virtue too often fails." The essay goes on to mention the causes of failure. The fact that many children die with small-pox during the first month of life, disproves, in the Dr.'s opinion, the assertion that a "failure may arise from vaccinating a child at too early an age."

The following he enumerates as legitimate causes of failure: "The virus may be used before it has undergone sufficient elaboration; vaccination is often performed by unprofessional persons, and not unfrequently, carelessly even by the professional man; the constitutional affection may be prevented by totally depriving the vesicle of its lymph; the progress of the vaccine vesicle may be modified by febrile action from whatever cause, or by the complication of all other affections, by which great constitutional disturbance is produced; and there are, also, certain idiosyncracies which secure an impunity, not only from the action of vaccina, but also from variola."

Does vaccine virus deteriorate in power in proportion to the number of times that it makes the circuit of the human body? Upon this subject the conclusions are as follows. That it does deteriorate "no good reason, and indeed no reason at all can be assigned." "As regards the effects of *variolo-vaccination*, which consists in inoculating the cow with the virus of variola, the resulting lymph being employed for the inoculation of man, it is questionable how far it may possess advantages, if any, over the ordinary current lymph." "Against the general adoption of this proposal, as well as to the immediate recurrence to the cow for the vaccinia, which last, among the variety of epizootic disorders affecting cattle, it is not easy to distinguish, several strong objections exist. When first taken from the cow, the lymph, in both instances, is often very acrid, producing local inflammation and glandular swellings; and, as regards the genuineness of the new stock of lymph, there will always be doubt until the undesirable experiment of variolous inoculation shall have been made. Moreover, years will often elapse before the true cow-pox, even in large dairies, can be found."

We also insert a quotation from Wilson's essays on Diseases of the Skin, which, if true, is both curious and valuable:

"We cannot here pass over an extraordinary statement made by Dr. Lichtenstein, as appears from Hufeland's Journal for 1841. The author, in a paper entitled, 'On the Sources from which Matter preservative against the Small-pox has been derived,' makes the remarkable assertion, that a pock undistinguishable from vaccinia, is produced in an unvaccinated person who is inoculated with the limpid lymph contained in the pustules caused by tartarized antimony. He further asserts that these pocks are equally protective against small pox, and that, like it, the lymph may be transmitted from individual to individual. From this source, he actually inoculated and re-inoculated thirty-one persons; and of those, notwithstanding all mingled freely with the infected during the epidemic prevalence of small-pox, not a single one took the disease."

Another quotation also is worthy of notice:

"As regards the general effect of vaccination," says Dr. John Davy, "in its influence both as affording protection from small-pox to a considerable extent, and mitigating its severity when not preventing the attack, the facts given are clear and satisfactory. *It is a curious circumstance, that the proportion of those who died after a second attack of small-pox, was, as has been already pointed out, greater than in the instances of those who had the disease after vaccination.*"

Is the protection increased by revaccination, and if so, under what circumstances? The discussion of this interrogatory comprises ten pages of the essay. We quote the condensed summary toward the close:

We are disposed to believe with Jenner, that when the system can be fully infected with the vaccine disease, a protection is afforded against the occurrence of small-pox, which will remain unimpaired with the continuance of life. But, at the same time, experience has established the fact, that among a given number apparently successfully vaccinated, there are some in whom, from the various and not well determined causes which interfere with the success of vaccination, a certain degree of susceptibility to variolous infection would seem to have been left unextinguished, and which appears to augment partially with the lapse of time. That re-vaccination exerts a powerful influence in diminishing these varioloid diseases, by giving to the system a new protection, has been abundantly proved by the statistics of re-vaccination furnished by the German states. As the mere admission of a possibility of a decline of the vaccine influence, shows the obvious necessity of re-vaccination, there remains no other question to be determined than the periods proper for the performance of the operation.

"Re-vaccination, in truth, promises us more than one advantage. Simple and harmless in its operation, contrary to variolous inoculation which serves to propagate the disease itself, it will determine, if the vaccinated person is susceptible of its influence, in the first place, the unprotected state of the system, and in the second, the future protection of the individual. But to determine precisely the length of time that the system is protected, is a question that does not admit of solution in the present state of our knowledge. From the preceding facts relating to this point, we are led to infer, more especially in considering the physiological changes which are known to be constantly occurring in the system, that the existence of this protecting influence has a variable period. *Let our object, therefoie, be to secure, at every period of life, the perfection of vaccination.*

"'You must bear in mind,' says Erasmus Wilson, Esq., 'that the greatest safety against small-pox that man can enjoy, is the possession of that modified constitution that succeeds to the fever of small-pox. Experience teaches that the amount of eruption is of little consequence, as much benefit flowing from the benign, as from the most confluent kind—from inoculated as from natural small-pox; and I have no hesitation in declaring that as much protection as small-pox can bestow, is derivable from perfect vaccination.'

"'I should advise,' he continues, 'that vaccination be repeated every seven or ten years. If the system receive not the inoculated virus, it may be regarded as protected, and no inconvenience results to the subject of operation. While, on the other hand, if the operation be successful, the inconvenience will be temporary, and trifling, but the advantages great.'

"The epidemics of small-pox, which have appeared in various parts of Italy during the last few years, have afforded Italian practitioners an excellent opportunity of studying many points connected with it. All the medical men, and M. Tommassini in particular, agree in stating that persons attacked were either adults or individuals who had been vaccinated for many years previously. When the disease occurred in children recently vaccinated, it was a simple varioloid or varicella. From the facts thus observed, the celebrated professor of Italy concludes that the preservative influence of vaccination lasts about ten or twelve years. Hence, he advises that re-vaccination should be had recourse to after the lapse of this period.—*Prov. Med. Jour.*, April 29, 1842, from *Il. Ra. Med.*

"In the *Annales D'Hygiène*, for July, 1837, Tome XVIII., there is an extended paper entitled '*Histoire d'une Epidémie de Variole, etc., par M. Charles Roesch*,' in which the following directions as regards re-vaccination, and of which we highly approve, are given:—

"1. To submit to vaccination all individuals who have not been vaccinated, even when they have had variola;

"2. To repeat the vaccination ten or twelve years after the first vaccination;

"3. If this re-vaccination does not prove successful, it will be necessary to repeat it from year to year until complete success shall follow;

"4. Should the re-vaccination prove entirely successful, the disposition to contract small-pox ought to be, for many subsequently, excessively feeble; but, notwithstanding this condition of the system, that is, an individual successfully re-vaccinated, a proper exercise of prudence would require, after ten or twelve years, a second re-vaccination.

"Now, this is a philosopher after own heart. We go upon the principle, *that the more you vaccinate the better.* We have re-vaccinated ourselves annually for the last ten or twelve years, but the year of our complete success has not yet come; and we regard ourselves, judging from the fact of repeated exposure, as entirely protected against the variolous poison. If individuals are successfully vaccinated in childhood, all facts would seem to prove that there is no necessity for re-vaccination before the tenth year of age; and the same data lead to the conclusion that the most suitable age is from the age of puberty to that of confirmed manhood. Our own opinion is that vaccination should be repeated at the age of 15 years or earlier, and again at 25. After this last period, as man seems to acquire, with the advancing years, an inaptitude to variola, there would seem to be no farther necessity for vaccination.

